# Community Characteristics and Performance of Phenanthrene-Degrading Microbial Consortia and Immobilized Composite Beads from Contaminated Sites

**DOI:** 10.3390/microorganisms14071465

**Published:** 2026-07-03

**Authors:** Langyue Chen, Zhenhua Zhao, Liling Xia, Zhirui Qin, Deqiang Chen

**Affiliations:** 1 Key Laboratory of Integrated Regulation and Resource Development on Shallow Lake of Ministry of Education, College of Environment, Hohai University, Nanjing 210098, China; 2 Department of Plant, Soil, and Microbial Sciences, Michigan State University, East Lansing, MI 48824, USA; 3 School of Computer & Software, Nanjing University of Industry Technology, Nanjing 210016, China

**Keywords:** polycyclic aromatic hydrocarbons (PAHs), phenanthrene, PAH-degrading microbial consortia, microbial community structure, immobilized composite beads, biodegradation

## Abstract

Although microbial remediation is a promising strategy for PAH pollution control, its field application remains a significant challenge. PAH-degrading microbial consortia were enriched from contaminated sites in Nanjing. High-throughput sequencing was applied to analyze the community structure and functional characteristics of bacteria and fungi, and the phenanthrene degradation performance of free consortia and sodium alginate-activated carbon-immobilized composite beads was systematically evaluated. Results showed that the distance from the pollution source was the key factor driving the differentiation of microbial community structure. For bacteria, sites closer to the pollution source showed significantly lower bacterial diversity and richness, while an opposite trend was observed for fungi. *Proteobacteria* (40–87%) and *Ascomycota* (51–88%) were the dominant phyla of bacterial and fungal communities, respectively. Despite significant differences in genus-level community composition among samples, the functional gene abundance related to PAHs metabolism was highly similar across all consortia. The immobilized composite beads achieved a significantly higher phenanthrene degradation efficiency (94.70–99.26%) compared with free consortia (65.84–85.78%). The embedding material had a significant effect on degradation performance, while nutrient sources showed no significant impact on the degradation efficiency. This study provides theoretical support for the application of immobilized microbial technology in PAH-contaminated site remediation.

## 1. Introduction

Polycyclic aromatic hydrocarbons (PAHs) are a class of persistent organic pollutants with fused aromatic ring structures, characterized by high lipophilicity, strong carcinogenicity, teratogenicity, mutagenicity, and bioaccumulation potential [1,2]. Due to their stable physicochemical properties, PAHs can persist in environmental media for a long time, and 16 PAH congeners have been listed as priority control pollutants by the U.S. Environmental Protection Agency [3]. Soil is the primary sink for PAHs in the environment, bearing more than 90% of the total environmental PAH load [4,5]. Many countries face severe PAH contamination, with total PAH emissions continuously rising with industrial development, and high-molecular-weight PAHs are the predominant species in surface soil [6,7]. Long-term exposure to PAH-polluted environments directly damages human health, and PAHs can also be biomagnified through the food chain, posing indirect health risks. Therefore, developing efficient remediation technologies for PAH-contaminated soil has become an urgent environmental issue.

Microbial remediation has attracted extensive attention for PAH pollution control due to its low cost, environmental friendliness, and low secondary pollution risk [8,9]. This technology removes PAHs mainly through microbial metabolic transformation, converting highly toxic PAHs into low-toxic or non-toxic small-molecule compounds. However, the field application of this technology is severely restricted: inoculated free-degrading bacteria are prone to loss, growth inhibition by indigenous microorganisms, and difficulty in adapting to complex field environmental conditions, resulting in unstable remediation efficiency [10].

Microbial immobilization technology can effectively address the above bottlenecks [11]. This technology fixes functional microorganisms in specific carrier materials, maintaining high biomass density, enhancing environmental stress resistance, and protecting exogenous degrading bacteria from the antagonism of indigenous microorganisms [12,13]. The embedding method is the most widely used immobilization strategy due to its simple operation, mild conditions, and minimal damage to microbial activity [14]. Sodium alginate, a natural polysaccharide-based embedding carrier, is extensively adopted in microbial immobilization for organic-contaminated soil remediation [15], has been repeatedly demonstrated to boost pollutant degradation efficiency when fabricated into composite carrier systems [16]. It was chosen as the basal embedding matrix in this work for its core strengths: superior biocompatibility that well maintains microbial metabolic activity, mild ionic gelation conditions with negligible cytotoxicity, and a porous network architecture that offers mechanical protection against environmental stress for immobilized cells while supporting efficient mass transfer of substrates and metabolites [15]. When combined with activated carbon, it can integrate the advantages of embedding and adsorption methods, further improving PAH enrichment and degradation efficiency [17].

Recent studies have increasingly focused on the application of immobilized microbial consortia for PAH remediation. For instance, microbial community responses to PAH stress have been investigated in various contaminated soils, revealing the importance of functional redundancy and community resilience [18]. Advances in carrier materials, such as biochar-alginate composites, have also been shown to enhance the stability and degradation performance of immobilized microorganisms [19]. More recently, a study developed a sodium alginate-immobilized microbial consortium (SA-IMC) system that achieved favorable PAH degradation performance in multiple aqueous matrices via process optimization with response surface methodology (RSM) and an artificial neural network (ANN) [20]. However, most existing studies, including the aforementioned process optimization work, focus on pure bacterial strains or artificially assembled consortia in aqueous systems, and reports on the community characteristics of PAH-degrading microbial consortia from actual contaminated sites remain limited. Furthermore, the performance of immobilized composite beads loaded with mixed degrading consortia from site-derived enrichments has not been systematically evaluated, and the differential responses of bacterial and fungal communities to pollution gradients in the same contaminated sites are poorly understood.

The overarching aim of this study was to comprehensively characterize site-derived phenanthrene-degrading microbial consortia and evaluate their performance when immobilized in composite beads for enhanced bioremediation. The specific objectives were to: (1) reveal the response of bacterial and fungal communities to PAH pollution stress in actual contaminated sites; (2) evaluate the phenanthrene degradation efficiency of immobilized composite beads loaded with site-derived degrading consortia; (3) clarify the effects of embedding materials and nutrient sources on the degradation performance of immobilized systems. This study provides a theoretical basis for the application of immobilized microbial technology in PAH-contaminated soil remediation.

## 2. Materials and Methods

### 2.1. Chemicals and Media

Phenanthrene (Phe, purity > 98%), acetonitrile, n-hexane, urea, sodium alginate, activated carbon, calcium chloride, and all inorganic reagents used in this study were of analytical grade, and purchased from Sinopharm Chemical Reagent Co., Ltd. (Shanghai, China).

Mineral Salt Medium (MSM) was used for bacterial enrichment and degradation experiments, containing (per liter of distilled water): 0.1 g of MgSO_4_, 0.67 g of NH_4_Cl, 0.99 g of K_2_HPO_4_, 1.0 g of KH_2_PO_4_, 0.02 g of CaCl_2_, 0.03 g of FeCl_3_, 0.025 g of NH_4_MoO_4_, 0.0005 g of CuSO_4_·5H_2_O, 0.0045 g of MnSO_4_, and 0.005 g of ZnSO_4_·7H_2_O. A 1000 mg/L phenanthrene stock solution was prepared with acetonitrile as the solvent. The liquid-selective MSM medium was formulated by spiking an appropriate volume of this stock solution into pre-autoclaved MSM to reach target phenanthrene concentrations, which were set as a gradient of 2–10 mg/L for the stepwise acclimation of degrading consortia. The pH of all media was adjusted to 7.0 prior to sterilization by autoclaving at 121 °C for 20 min [21].

### 2.2. Soil Sampling

Four surface soil samples (0–20 cm depth) were collected from the Liuhe Development Zone, Nanjing, China, based on historical contamination records and preliminary surveys indicating different levels of PAH pollution. For each sampling site, five subsamples were obtained via the five-point sampling method and fully homogenized to generate one representative composite sample, with approximately 500 g of fresh soil retained for each site. The sampling sites were: (1) interior of Yangzi Petrochemical Plant (labeled PB1); (2) soil above the oil storage tank of a nearby Sinopec gas station (PB2); (3) meadow outside Yangzi Petrochemical Plant (PB3); (4) meadow outside the Sinopec gas station (PB4). After sampling, surface residues and stones were removed, and soil samples were sealed in sterile plastic self-sealing bags, numbered, and transported to the laboratory immediately. Upon arrival, all soil samples underwent aseptic pretreatment in a laminar flow cabinet: fine impurities and plant residues were further removed under sterile conditions, and the samples were passed through a sterilized 2 mm standard soil sieve and thoroughly homogenized. All samples were stored at 4 °C after aseptic treatment for subsequent enrichment experiments.

### 2.3. Enrichment and Screening of Phenanthrene-Degrading Microbial Consortia

For each soil sample, 20 g of soil was soaked in 200 mL of sterile distilled water for 24 h with thorough stirring, then shaken at 30 °C and 120 r/min for 3 d. The purpose of this soaking step was to release indigenous microorganisms from the soil matrix into the aqueous phase through physical dispersion and mild shaking, without introducing exogenous nutrients that could precondition the community prior to phenanthrene enrichment. Although distilled water causes temporary osmotic stress, the short duration (24 h static + 3 d shaking) minimized its impact, and the subsequent transfer to MSM with phenanthrene ensured that the enriched consortia were specifically selected for phenanthrene-degrading capacity. The supernatant was inoculated into liquid selective MSM medium with an initial phenanthrene concentration of 2 mg/L for enrichment culture.

Gradient acclimation was performed with increasing phenanthrene concentration: the bacterial suspension was transferred to fresh selective MSM medium every 7 d, with a phenanthrene concentration gradient of 2 mg/L per transfer (sequentially set to 2, 4, 6, 8, and 10 mg/L). The acclimation process was repeated until visible bacterial growth was observed in the medium. To prepare the working microbial suspension for subsequent immobilization and biodegradation assays, the final acclimated culture (obtained at a phenanthrene concentration of 10 mg/L with visible microbial growth) was centrifuged at 6000 r/min for 10 min to harvest bacterial pellets. Harvested pellets were washed twice with sterile 0.85% (*w*/*v*) normal saline to remove residual culture medium and phenanthrene, followed by resuspension in fresh sterile MSM to a standardized optical density at 600 nm (OD_600_ = 1.0). This standardization step ensured consistent inoculation biomass across all treatment groups.

Separately, an aliquot of the final acclimated culture was centrifuged to collect cell pellets for high-throughput sequencing. All prepared working suspensions were stored at 4 °C prior to use.

All enrichment procedures were performed under sterile conditions.

### 2.4. High-Throughput Sequencing and Bioinformatics Analysis

Total genomic DNA from the enriched microbial consortia was extracted using the OMEGA Soil DNA Extraction Kit, with three extraction replicates for each sample. The integrity of extracted DNA was verified via 1% agarose gel electrophoresis, and DNA purity and concentration were determined using a NanoDrop 2000 spectrophotometer (Thermo Fisher Scientific, Waltham, MA, USA).

PCR amplification of the bacterial 16S rRNA gene V3-V4 region was performed using the primer pair 338F (5′-ACTCCTACGGGAGGCAGCAG-3′) and 806R (5′-GGACTACHVGGGTWTCTAAT-3′), and the fungal ITS rRNA gene region was amplified using the primers ITS1F (5′-CTTGGTCATTTAGAGGAAGTAA-3′) and ITS2R (5′-GCTGCGTTCTTCATCGATGC-3′). The PCR reactions were conducted under the following conditions: initial denaturation at 95 °C for 3 min, followed by 27 cycles of denaturation at 95 °C for 30 s, annealing at 55 °C for 30 s, and extension at 72 °C for 45 s, with a final extension at 72 °C for 10 min. The amplified PCR products were purified using the AxyPrep DNA Gel Extraction Kit (Axygen Biosciences, Union City, CA, USA) according to the manufacturer’s instructions. Purified amplicons were then sequenced on the Illumina MiSeq platform (Illumina, San Diego, CA, USA) using the MiSeq Reagent Kit v3 (600-cycle) for paired-end (2 × 300 bp) sequencing.

Raw sequencing data were demultiplexed and quality-filtered using Trimmomatic (v0.39) to remove adapter sequences and low-quality reads (average quality score < 20 over a 50 bp sliding window). High-quality sequences were then clustered into Operational Taxonomic Units (OTUs) at a 97% similarity threshold using Usearch software (v11.0). Alpha diversity indices (Chao, Ace, Shannon, Simpson, Coverage) were calculated to reflect the richness and diversity of microbial communities. Hierarchical clustering analysis, Bray–Curtis distance heatmap, and Principal Coordinates Analysis (PCoA) were performed to analyze inter-sample differences in community structure. Functional prediction of bacterial communities was conducted using PICRUSt2 (Phylogenetic Investigation of Communities by Reconstruction of Unobserved States 2), based on the Kyoto Encyclopedia of Genes and Genomes (KEGG) and Clusters of Orthologous Groups (COG) databases. The OTU-based clustering approach with a 97% similarity threshold was selected because it remains a widely accepted and standardized method for cross-study comparability, and its robustness in capturing major community variation in enrichment cultures has been well-established. This threshold effectively balances computational efficiency and biological relevance for the diversity and functional analyses performed in this study, which focus on community-level patterns rather than strain-level resolution. The data processing was performed using the Meiji Bio’s cloud platform.

### 2.5. Preparation of Immobilized Composite Beads

Sodium alginate-activated carbon immobilized composite beads were prepared by the embedding method: briefly, 1 g of activated carbon powder was added to 50 mL of acclimated bacterial suspension, and the mixture was shaken at 30 °C and 120 r/min for 24 h for adsorption. After centrifugation to collect bacterial pellets, the pellets were mixed with 20 mL of 2% (*w*/*v*) sodium alginate solution, and nutrients were added for bacterial activation, either a 1:1 mixture of urea and dried branch powder or an MSM nutrient salt solution.

The homogeneous mixture was slowly dropped into 600 mL of 2% (*w*/*v*) CaCl_2_ solution using a sterile syringe and then cross-linked and hardened at room temperature for 8 h. The prepared spherical immobilized beads (2–3 mm in diameter) were washed twice with sterile water and stored at 4 °C in sterile closed containers (50 mL centrifuge tubes) submerged in sterile distilled water for subsequent degradation experiments. The dosages of sodium alginate, activated carbon, and bacterial suspensions (standardized to OD_600_ = 1.0) were consistent across all groups to ensure a comparable initial biomass input for degradation comparisons.

### 2.6. Phenanthrene Biodegradation Assays

Batch degradation experiments were carried out in 50 mL colorimetric tubes containing 40 mL of a 10 mg/L phenanthrene solution, incubated at 30 °C with shaking at 120 r/min. Five treatment groups were set for each microbial consortium sample, with three technical replicates for all groups:

- Group A: Immobilized composite beads with urea and dried branch mixture as nutrients;
- Group B: Immobilized composite beads with MSM nutrient salt solution as nutrients;
- Group C: Sterile sodium alginate-activated carbon beads without degrading consortia (adsorption control);
- Group D: Activated carbon pellets adsorbed with degrading consortia (no sodium alginate embedding);
- Group E: Only centrifuged wet bacterial pellets (free consortium control).

The dosages of sodium alginate, activated carbon, and bacterial suspensions (standardized to OD_600_ = 1.0) were consistent across all groups to ensure a comparable initial biomass input for degradation comparisons. The phenanthrene concentration was detected once per day for the first 7 d and every other day from day 8 to day 12, with a total of 10 detection time points for each group.

### 2.7. Detemination of Phenanthrene Concentration

The concentration of phenanthrene in the solution was determined by Agilent 1100 HPLC (Agilent, Santa Clara, CA, USA) with fluorescence and UV-adsorption detectors. The reversed-phase C18 column (Agilent ZORBAX Eclipse XDB-C18, 250 mm × 4.6 mm × 5 μm) was employed as the stationary phase. The mixed solution of acetonitrile and ultrapure water (75:25, *v*/*v*) was regarded as the mobile phase at a speed of 1 mL/min. Phenanthrene was quantified by employing external standard solutions obtained from Ehrenstorfer in Augsburg, Germany. Detective wavelength with FLD signal (Ex/Em = 257/380 nm). The phenanthrene degradation efficiency was calculated as follows:

(1) $$Degradation\;efficiency\;(\%)=\frac{m_{0}-m_{1}}{m_{0}}\times 100\%,$$

where *m*_1_ is the residual mass after incubation (mg), and *m*_0_ is the initial mass of phenanthrene (mg), with the adsorption control group (Group C) used to correct for non-biological loss of phenanthrene (including volatilization and physical adsorption).

### 2.8. Analytical Methods

All degradation experiments were performed with three technical replicates for each treatment group, and results are expressed as mean ± standard deviation (SD). Statistical comparisons between groups were performed using one-way analysis of variance (ANOVA) followed by Tukey’s post hoc test, with a significance level of *p* < 0.05. All statistical analyses were conducted using SPSS Statistics (version 26.0, IBM Corp., Armonk, NY, USA).

## 3. Results

### 3.1. Alpha Diversity and Inter-Sample Variation in Microbial Communities

The Coverage index of all samples ranged from 0.945 to 0.987, indicating that the sequencing results could reliably reflect the actual composition of the microbial communities in the samples (Table 1) and that the sequencing depth was sufficient.

For bacterial communities, the Chao, Ace, and Shannon indices of PB3 and PB4 (sites far from the pollution source) were significantly higher than those of PB1 and PB2 (sites close to the pollution source). PB4 had the highest bacterial diversity, while PB2 had the lowest. This result suggests that higher pollution levels may exert selective pressure that reduces bacterial diversity and richness. For fungal communities, the diversity indices of PB1 and PB2 were significantly higher than those of PB3 and PB4, with PB2 showing the highest fungal diversity and PB3 the lowest. The results indicated that the distance from the pollution source had opposite effects on the diversity of bacterial and fungal communities: closer proximity to the pollution source reduced bacterial diversity but increased fungal diversity, indicating a differential response of bacteria and fungi to PAH stress.

**Figure 1:**
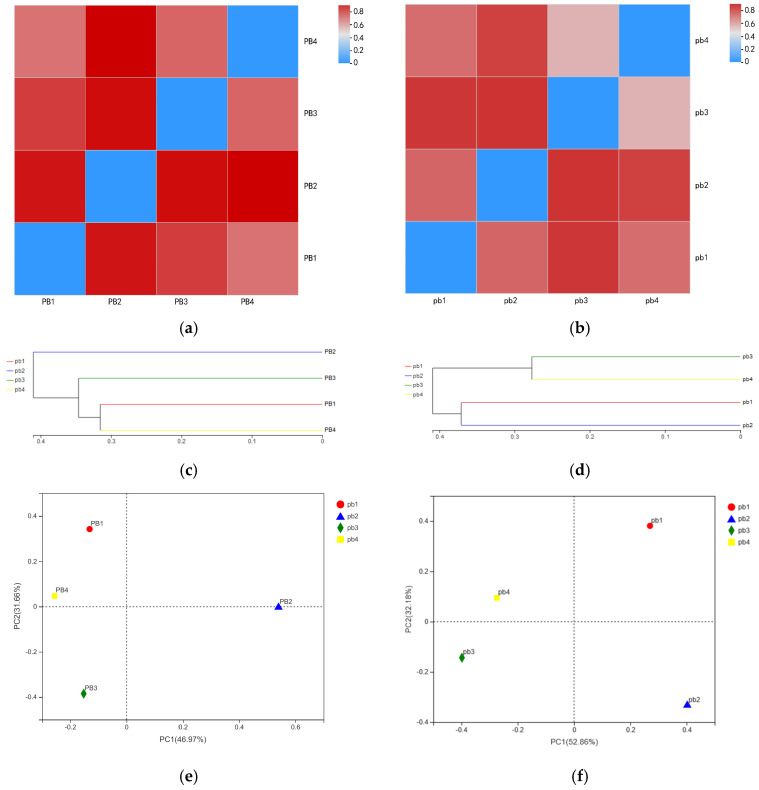
Bray–Curtis distance heatmap, Hierarchical clustering tree, and PCoA analysis of bacterial and fungal communities at the genus level. (**a**,**c**,**e**) Bacterial communities: (**a**) Bray–Curtis distance heatmap; (**c**) Hierarchical clustering tree; (**e**) PCoA analysis. (**b**,**d**,**f**) Fungal communities: (**b**) Bray–Curtis distance heatmap; (**d**) Hierarchical clustering tree; (**f**) PCoA analysis.

Inter-sample difference analysis was performed via hierarchical clustering, Bray–Curtis distance heatmap, and Principal Coordinates Analysis (PCoA) (Figure 1).

The results consistently showed that the bacterial community of PB2 was significantly differentiated from the other three samples, while PB1, PB3, and PB4 had relatively high similarity, suggesting a distinct community structure under higher pollution stress. For fungal communities, PB1 and PB2 were clustered into one group, and PB3 and PB4 were clustered into another group, with significant differentiation between the two groups, further indicating the influence of pollution gradients on community composition.

### 3.2. Taxonomic Composition of Phenanthrene-Degrading Microbial Communities

**Figure 2:**
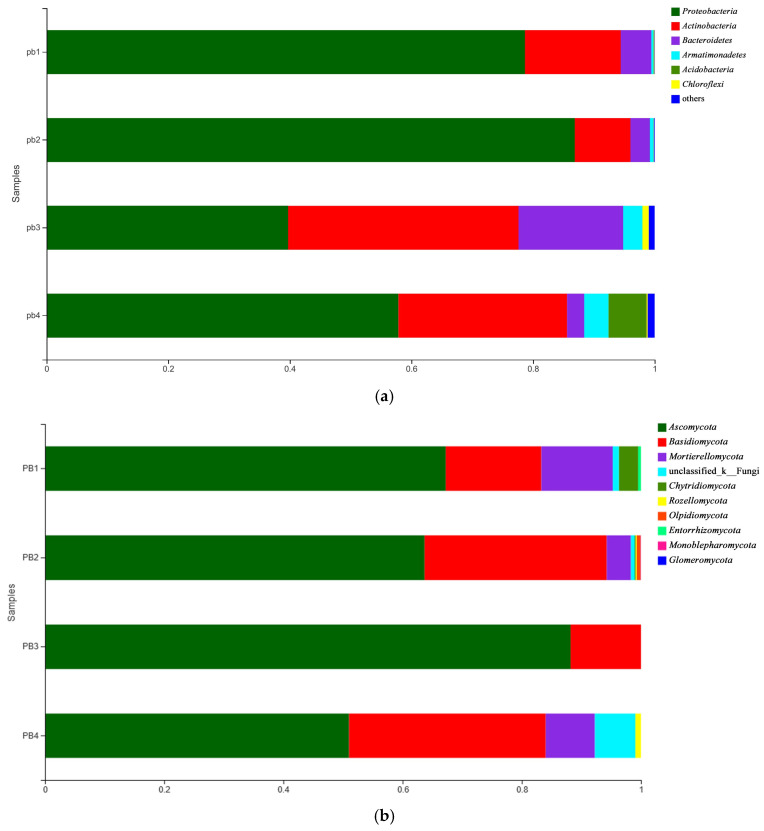
Community structure bar plot of bacterial (**a**) and fungal (**b**) communities at the phylum level.

The community structure at the phylum level was analyzed, and the results are shown in Figure 2a (bacteria) and Figure 2b (fungi). At the phylum level, the dominant bacterial phyla were consistent across all samples, mainly including *Proteobacteria*, *Actinobacteria*, *Bacteroidetes*, *Armatimonadetes*, *Acidobacteria*, and *Chloroflexi*. Among them, *Proteobacteria* had the highest relative abundance, ranging from 40% to 87% (highest in PB2), followed by *Actinobacteria* (9–38%) and *Bacteroidetes* (3–17%), indicating their dominant role in PAH degradation processes. The dominant fungal phyla were mainly *Ascomycota*, *Basidiomycota*, *Mortierellomycota*, *Unclassified_k_Fungi*, *Chytridiomycota*, and *Rozellomycota*. *Ascomycota* were the most dominant fungal phylum, with a relative abundance of 51–88% (highest in PB3), followed by *Basidiomycota* (12–33%) and *Mortierellomycota* (4–12%), suggesting their ecological importance in contaminated environments.

**Figure 3:**
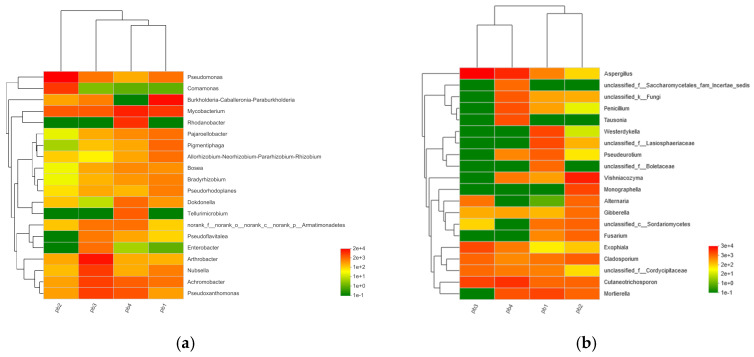
Species abundance heatmap of bacterial (**a**) and fungal (**b**) communities at the genus level.

**Figure 4:**
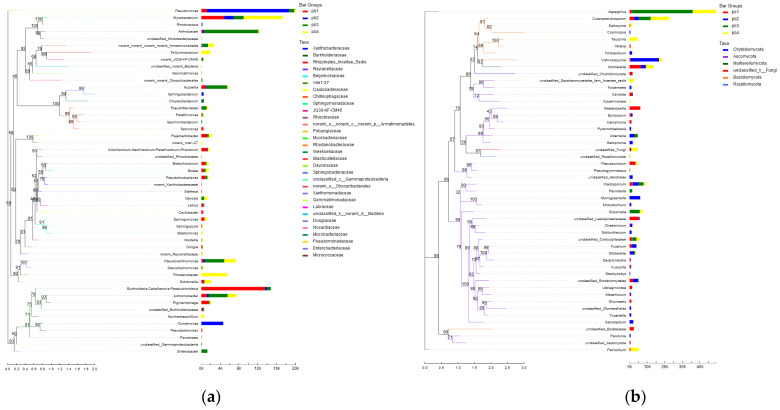
Phylogenetic tree of bacterial (**a**) and fungal (**b**) communities at the genus level based on enriched consortia from the four sampling sites.

At the genus level, the microbial community structure showed more significant differences among samples, as shown in the species abundance heatmap (Figure 3) and phylogenetic tree (Figure 4).

The dominant genera of bacterial and fungal communities in different samples are shown in Figure 4. For bacteria, *Burkholderia-Caballeronia-Paraburkholderia* was dominant in PB1; *Pseudomonas* and *Comamonas* were highly abundant in PB2; *Arthrobacter*, *Nubsella*, *Achromobacter*, and *Pseudoxanthomonas* were dominant in PB3; *Rhodanobacter* was the most abundant in PB4. *Mycobacterium* maintained high abundance in all samples, indicating that it may serve as a core PAH-degrading genus across sites.

For fungi, *Westerdykella*, *unclassified_f_Lasiosphaeriaceae*, and *Mortierella* were dominant in PB1; *Vishniacozyma* and *Monographella* were highly abundant in PB2; *Aspergillus* and *Exophiala* were dominant in PB3; *Aspergillus* and *Mortierella* were the main genera in PB4. *Cutaneotrichosporon* and *Cladosporium* had high abundance in all samples, suggesting their widespread distribution in PAH-contaminated environments.

Notably, while many of these genera have been previously reported as PAH-degraders in the literature, the presence of a genus in the enriched consortium does not confirm that all members of that genus possess phenanthrene-degrading capability; some community members may be cross-feeders utilizing metabolites produced by primary degraders.

### 3.3. Functional Prediction of Microbial Communities

**Figure 5:**
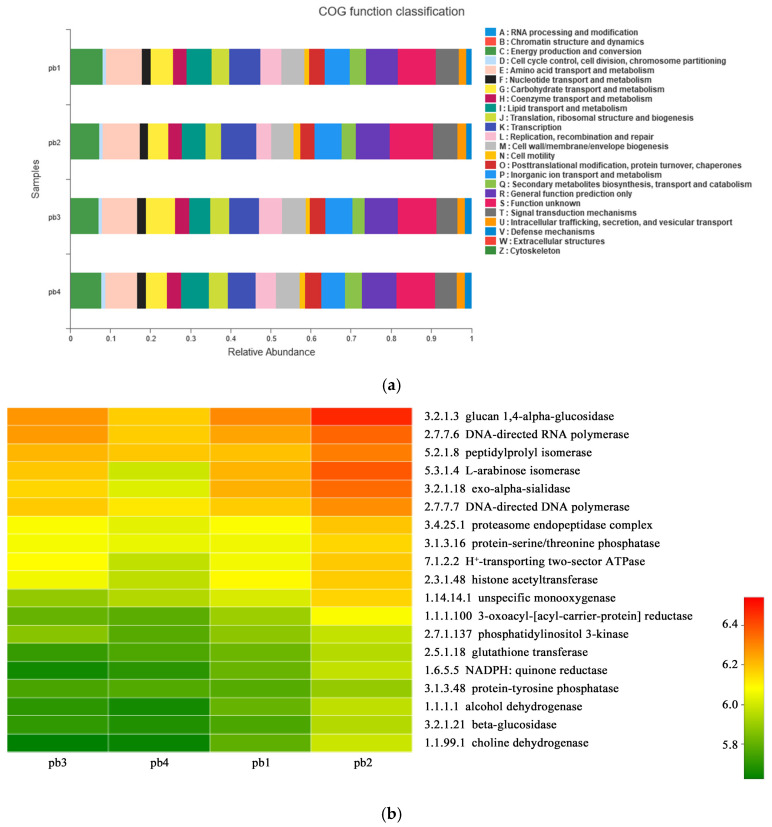
COG functional classification bar plot of bacterial communities (**a**) and KEGG functional heatmap of fungal communities (**b**).

Functional prediction of the microbial communities was performed using PICRUSt, and the results are shown in Figure 5a (bacterial COG functional classification) and Figure 5b (fungal KEGG functional heatmap).

COG functional classification and KEGG functional prediction showed that, despite the significant differences in community composition among the four samples, their functional profiles were highly similar, with only minor differences in functional gene abundance, indicating a certain degree of functional redundancy among microbial communities. The main functional categories were related to microbial growth, reproduction, and metabolism, including energy production and conversion; amino acid transport and metabolism; carbohydrate transport and metabolism; lipid transport and metabolism; and replication, recombination and repair. Among them, genes with unknown functions had the highest relative abundance, followed by genes associated with amino acid transport and metabolism, and general function prediction only, suggesting the presence of unexplored metabolic potential. It should be noted that PICRUSt-based predictions reflect metabolic potential rather than actual gene expression, and specific ring-hydroxylating dioxygenase genes characteristic of bacterial phenanthrene degradation were not directly detected in this analysis.

### 3.4. Phenanthrene Degradation Performance of Immobilized Composite Beads

**Figure 6:**
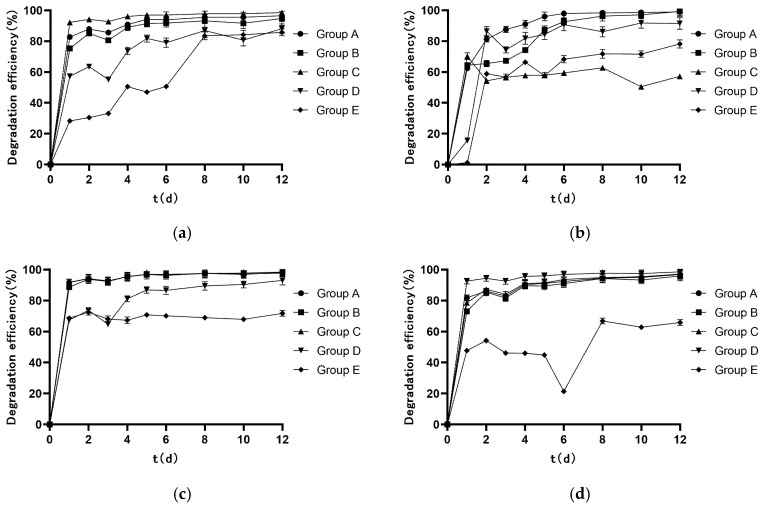
Phenanthrene degradation characteristics of different treatment groups for PB1 (**a**), PB2 (**b**), PB3 (**c**), and PB4 (**d**). Group A: Immobilized composite beads with urea and dried branch mixture as nutrients; Group B: Immobilized composite beads with MSM nutrient salt solution as nutrients; Group C: Sterile sodium alginate-activated carbon beads (adsorption control); Group D: Activated carbon pellets adsorbed with degrading consortia (no sodium alginate embedding); Group E: Free degrading consortia (control).

The phenanthrene degradation characteristics of different treatment groups for each sample are shown in Figure 6.

The degradation results showed that both immobilized composite beads and free consortia could degrade phenanthrene in aqueous solution, while the immobilized beads achieved a significantly faster degradation rate and higher final degradation efficiency, indicating the effectiveness of immobilization in enhancing biodegradation performance. The phenanthrene degradation efficiency of the immobilized beads reached a stable plateau after 7 d of incubation, while the free consortia showed a longer lag period and a slower degradation rate, suggesting a lower adaptability of free microorganisms under the experimental conditions.

**Figure 7:**
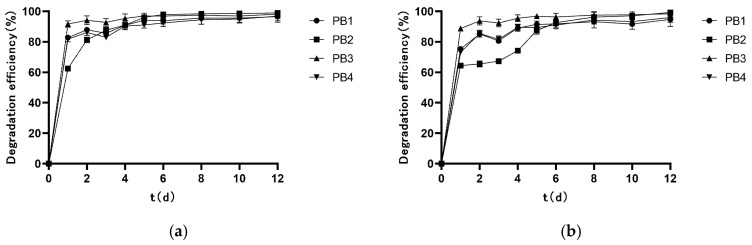
Phenanthrene degradation characteristics of immobilized beads from Group A (**a**) and Group B (**b**). Group A: Immobilized beads with urea and dried branch mixture as nutrients; Group B: Immobilized beads with MSM nutrient salt solution as nutrients.

For Group A (urea and dried branch mixture as nutrients), the final phenanthrene degradation efficiencies of PB1, PB2, PB3, and PB4 were 96.59%, 99.00%, 98.20%, and 96.86%, respectively. For Group B (MSM nutrient salt solution as nutrients), degradation efficiencies were 94.70%, 99.26%, 98.47%, and 95.94%, respectively. The difference in degradation efficiency between the two groups for the same sample was less than 2%, indicating that different nutrient sources for bacterial activation had no significant effect on the final phenanthrene degradation efficiency of the immobilized beads. The degradation characteristics of the two nutrient source groups are shown in Figure 7.

Compared with single-adsorption or embedding materials, the sodium alginate-activated carbon composite immobilized beads showed superior degradation performance. The sterile adsorption control group (Group C) had a certain phenanthrene adsorption capacity, but the final removal rate was lower than that of the groups inoculated with degrading consortia. The activated carbon adsorption group (Group D) had a lower degradation efficiency than the composite immobilized bead groups, indicating that the combination of adsorption and immobilization enhanced degradation efficiency.

### 3.5. Phenanthrene Degradation Performance of Free Consortia from Different Sampling Sites

The phenanthrene degradation efficiencies of free consortia from the four samples are shown in Table 2. The degradation efficiencies ranged from 65.84% to 85.78%, with an average value of 75.39%. PB1 had the highest degradation efficiency (85.78%), followed by PB2 (78.19%), PB3 (71.77%), and PB4 (65.84%). The results showed that the degrading consortia screened from sites closer to the pollution source had better phenanthrene degradation performance, suggesting long-term adaptation to pollutant stress. When normalized to the initial biomass input (OD_600_ = 1.0 for both free and immobilized groups), the immobilized composite beads still demonstrated significantly higher specific degradation activity, with degradation efficiencies of 94.70–99.26% compared to 65.84–85.78% for free consortia, representing a 10–30% increase in activity per unit of biomass. This enhancement is attributed to the protective effect of the immobilization matrix, which maintains higher viable cell counts and metabolic activity throughout the degradation period. Compared with free consortia, the immobilized composite beads increased the phenanthrene degradation efficiency by 10–30%, further confirming the advantage of immobilization technology.

## 4. Discussion

In this study, we enriched phenanthrene-degrading microbial consortia from four sampling sites at different distances from pollution sources and found that the distance from the pollution source was the key factor driving the differentiation in microbial community structures, highlighting the importance of pollution gradients in shaping microbial ecology. For bacterial communities, sites closer to the pollution source showed significantly lower diversity and richness. This result can be attributed to the directional selection pressure of high concentrations of PAHs and petroleum hydrocarbons: bacterial species that cannot adapt to high pollution stress cannot survive, leading to a decrease in bacterial diversity [22], which is consistent with environmental filtering theory. In contrast, fungal diversity was higher in sites closer to the pollution source, indicating that fungi may contain more degrading species that are adaptable to PAH-contaminated environments, and can be enriched under the induction of pollutants [23], suggesting stronger tolerance or metabolic versatility of fungi under stress conditions.

*Proteobacteria* were the dominant bacterial phylum in all enriched consortia, which is widely reported as the dominant phylum in PAH-contaminated environments, containing a large number of well-documented PAH-degrading genera such as *Pseudomonas*, *Comamonas*, and *Burkholderia* [24]. *Mycobacterium*, which maintained high abundance in all samples, is also a typical PAH-degrading genus capable of utilizing high-molecular-weight PAHs as the sole carbon source [9]. For fungi, *Ascomycota* was the dominant phylum, and *Aspergillus*, the dominant genus in PB3 and PB4, has been widely confirmed to have strong PAH degradation capacity [25]. Despite the significant differences in genus-level community composition among samples, the functional profiles related to carbon metabolism and PAH degradation were highly similar, indicating functional redundancy among microbial communities. It should be noted that the taxonomic composition of the enriched consortia reflects the presence of various genera, but not all members of these genera are necessarily active phenanthrene degraders. Some community members may contribute indirectly through metabolic cross-feeding or co-metabolism, which is a common phenomenon in mixed microbial consortia [26,27]. Future studies using stable isotope probing (SIP) or single-cell analysis are needed to identify the active degraders within the consortia.

The batch degradation experiments showed that the sodium alginate-activated carbon-immobilized composite beads significantly improved phenanthrene degradation efficiency compared with free consortia, with a 10–30% increase in degradation efficiency. This improvement can be attributed to three main reasons: first, the porous network structure of the sodium alginate gel provides a suitable microenvironment for microbial growth, maintaining a high biomass density; second, the embedding matrix protects degrading microorganisms from adverse environmental impacts, improving their stress resistance; third, the activated carbon in the composite beads can adsorb and enrich phenanthrene in the solution, increasing the contact probability between pollutants and degrading microorganisms, thus accelerating the degradation process [17,28]. The composite beads integrate the advantages of embedding and adsorption methods, which is consistent with previous studies on immobilized microbial technology for organic pollutant degradation [29].

In addition, different nutrient sources for bacterial activation showed no significant effect on the final degradation efficiency of the immobilized beads. Interestingly, within the immobilized bead system, the two different nutrient sources (urea + dried branch powder vs. MSM nutrient salt solution) resulted in nearly identical final phenanthrene degradation efficiencies (difference < 2%), indicating that the immobilized matrix itself provides a stable and protective microenvironment that supports microbial metabolic activity regardless of the specific nutrient formulation. This flexibility in nutrient selection is advantageous for practical field applications, as inexpensive and locally available nutrient sources can be used without compromising degradation performance. The degrading consortia from sites closer to the pollution source showed better degradation performance, which is due to the long-term directional induction of pollutants, making the microbial communities more adaptable to PAH pollution and having higher metabolic activity for PAHs [30].

Overall, the improved degradation performance observed in this study is broadly consistent with recent evidence that sodium alginate-immobilized microbial consortia hold promise for PAH remediation [20]. Nevertheless, whereas prior work has centered on data-driven process parameter optimization in aqueous matrices, the present work extends this research scope by characterizing the divergent responses of in situ enriched bacterial and fungal communities along PAH pollution gradients, and by evaluating the degradation performance of site-derived consortia immobilized in sodium alginate-activated carbon composite beads for soil remediation applications. These findings may offer complementary insights to support the further development of immobilized bioremediation toward field implementation.

Several limitations of this study should be acknowledged. First, actual phenanthrene concentrations at the sampling sites were not measured at the time of collection, and the pollution gradient was inferred based on the distance from the pollution source and historical contamination records. Second, while PICRUSt-based functional prediction revealed metabolic adaptation signatures, it does not directly detect specific ring-hydroxylating dioxygenase genes (e.g., *nahAc*, *phdA*, or *PAH-RHDα*) that are characteristic of bacterial phenanthrene degradation, reflecting the inherent limitation of 16S rRNA-based predictive approaches. Third, direct extraction of phenanthrene from the immobilized beads was not performed at the end of the experiments, so the sorbed versus biodegraded fractions within the beads could not be quantitatively distinguished. Fourth, the degradation experiments were conducted only in aqueous systems, and the remediation performance of the immobilized beads in PAH-contaminated soil requires further verification. Fifth, only phenanthrene was used as the target pollutant, and the degradation capacity of the consortia for high-molecular-weight PAHs and complex PAH mixtures remains unexplored. Sixth, the enriched consortia were used for immobilization without isolation and purification of the core functional strains, limiting our ability to elucidate the degradation mechanisms and metabolic pathways at the strain level. Future studies should incorporate quantitative soil PAH analysis; metagenomic or functional gene-targeted approaches; direct extraction of phenanthrene from immobilized beads to distinguish sorbed and biodegraded fractions for comprehensive mass-balance analysis; in situ soil remediation experiments; and strain isolation to address these gaps. Regarding the use of acetonitrile as the solvent, its final concentration in the culture medium ranged from 0.2% to 1.0% (*v*/*v*) during enrichment and degradation; at this level, acetonitrile is unlikely to serve as a significant carbon source or cause substantial toxicity to the enriched consortia [31,32], as further supported by the successful enrichment and high degradation performance observed in this study.

## 5. Conclusions

In this study, phenanthrene-degrading microbial consortia were enriched from four sampling sites with different pollution levels, and the community characteristics and immobilized degradation performance were systematically investigated, providing insights into the ecological response and application potential of site-derived microbial consortia. The main conclusions are as follows:

1. The distance from the pollution source was the main factor driving the differentiation of microbial community structure. Closer proximity to the pollution source resulted in lower diversity and richness of bacterial communities, but higher diversity and richness of fungal communities, indicating distinct adaptive responses of bacteria and fungi to PAH contamination.
2. *Proteobacteria* and *Ascomycota* were the dominant phyla of bacterial and fungal communities, respectively. Although the genus-level community composition varied significantly among samples, the functional gene abundance related to PAH metabolism was highly similar across all consortia, suggesting a certain degree of functional redundancy in PAH-degrading microbial communities.
3. The sodium alginate-activated carbon immobilized composite beads achieved a phenanthrene degradation efficiency of 94.70–99.26%, which was 10–30% higher than that of free consortia. The composite embedding material significantly improved the degradation efficiency, while different nutrient sources for bacterial activation had no significant effect on the final degradation performance, demonstrating the stability and robustness of the immobilized system.
4. The degrading consortia from sites closer to the pollution source had better phenanthrene degradation performance, but the overall PAH degradation function of the consortia from different sites had little difference, indicating that long-term pollutant exposure enhances degradation capacity while maintaining functional consistency across communities.

## Figures and Tables

**Table 1:** Alpha diversity indices of bacterial and fungal communities from different samples.

	Sample	Sobs	Chao	Ace	Shannon	Simpson	Coverage
Bacterial communities	PB1	48	50.00	52.411	2.346	0.203	0.987
	PB2	47	50.75	52.207	1.423	0.429	0.978
	PB3	75	76.25	78.205	2.514	0.147	0.986
	PB4	92	105.00	103.245	2.750	0.123	0.958
Fungal communities	PB1	77	78.50	79.599	3.563	0.047	0.945
	PB2	214	214.20	214.690	4.041	0.041	0.972
	PB3	21	21.00	21.744	1.574	0.373	0.982
	PB4	45	48.00	47.172	2.831	0.089	0.945

Note: Chao and Ace indices reflect community richness; Shannon and Simpson indices reflect community diversity; Coverage index reflects sequencing depth. PB1: inside Yangzi Petrochemical Plant; PB2: above Sinopec gas station oil storage tank; PB3: meadow outside Yangzi Petrochemical Plant; PB4: meadow outside Sinopec gas station.

**Table 2:** Phenanthrene degradation efficiencies of free consortia from different samples.

Sample	Phenanthrene Degradation Efficiency	Sampling Site Description
PB 1	85.78%	Inside Yangzi Petrochemical Plant
PB 2	78.19%	Above Sinopec gas station oil storage tank
PB 3	71.77%	Meadow outside Yangzi Petrochemical Plant
PB 4	65.84%	Meadow outside Sinopec gas station

Note: The degradation efficiency is the final value after 12 d of incubation.

## Data Availability

The data presented in this study are available on request from the corresponding author due to requirements for data confidentiality.

## Abbreviations

The following abbreviations are used in this manuscript:

COG	Clusters of Orthologous Groups
DNA	Deoxyribonucleic Acid
FLD	Fluorescence Detector
HPLC	High Performance Liquid Chromatography
KEGG	Kyoto Encyclopedia of Genes and Genomes
MSM	Mineral Salt Medium
OTUs	Operational Taxonomic Units
PAHs	Polycyclic Aromatic Hydrocarbons
PCoA	Principal Coordinates Analysis
PCR	Polymerase Chain Reaction
Phe	Phenanthrene
PICRUSt	Phylogenetic Investigation of Communities by Reconstruction of Unobserved States
RNA	Ribonucleic Acid
rRNA	Ribosomal RNA
